# Analysis Treatment Guideline versus Clinical Practice Protocol in Patients Hospitalized due to Heart Failure

**DOI:** 10.5935/abc.20160018

**Published:** 2016-03

**Authors:** Alessandra da Graça Corrêa, Marcia Makdisse, Marcelo Katz, Thamires Campos Santana, Paula Kiyomi Onaga Yokota, Tatiana de Fatima Gonçalves Galvão, Fernando Bacal

**Affiliations:** 1 Hospital Israelita Albert Einstein, São Paulo, SP - Brasil; 2 Instituto do Coração da Faculdade de Medicina da USP, São Paulo, SP - Brasil

**Keywords:** Heart Failure / therapy, Inpatients, Protocol, Quality Indicators, Health Care

## Abstract

**Background:**

Despite the availability of guidelines for treatment of heart failure (HF), only a few studies have assessed how hospitals adhere to the recommended therapies.

**Objectives:**

Compare the rates of adherence to the prescription of angiotensin-converting enzyme inhibitor or angiotensin II receptor blockers (ACEI/ARB) at hospital discharge, which is considered a quality indicator by the Joint Commission International, and to the prescription of beta-blockers at hospital discharge, which is recommended by national and international guidelines, in a hospital with a case management program to supervise the implementation of a clinical practice protocol (HCP) and another hospital that follows treatment guidelines (HCG).

**Methods:**

Prospective observational study that evaluated patients consecutively admitted to both hospitals due to decompensated HF between August 1st, 2006, and December 31st, 2008. We used as comparing parameters the prescription rates of beta-blockers and ACEI/ARB at hospital discharge and in-hospital mortality.

**Results:**

We analyzed 1,052 patients (30% female, mean age 70.6 ± 14.1 years), 381 (36%) of whom were seen at HCG and 781 (64%) at HCP. The prescription rates of beta-blockers at discharge at HCG and HCP were both 69% (p = 0.458), whereas those of ACEI/ARB were 83% and 86%, respectively (p = 0.162). In-hospital mortality rates were 16.5% at HCP and 27.8% at HCG (p < 0.001).

**Conclusion:**

There was no difference in prescription rates of beta-blocker and ACEI/ARB at hospital discharge between the institutions, but HCP had lower in-hospital mortality. This difference in mortality may be attributed to different clinical characteristics of the patients in both hospitals.

## Introduction

Heart failure (HF) is the most frequent cause of hospitalization due to circulatory system diseases in individuals older than 20 years in Brazil. It represents 3% of the total hospital admissions and 23% of the hospital admissions due to cardiovascular diseases.^1^ In the United States alone, annual estimates indicate 500 thousand new HF cases generating an approximate cost of 34.8 million dollars.^2-4^

Patients with HF have a substantial risk of recurrent acute exacerbations and up to 50% of those who are discharged from the hospital are readmitted within 6 months. Treatment advances have increased the life expectancy of patients with HF. However, the mortality rate associated with the disease is still high, and approximately 12% of the patients die within 30 days, and 33% within 1 year after the first hospitalization.^2^

Considering this scenario and seeking to improve the care of these patients, quality accreditation organizations such as the *Centers for Medicare and Medicaid Services* (CMS) along with the *Joint Commission International* (JCI) have developed metrics to assess how hospitals perform in HF treatment based on four quality indicators: 1) record of assessment of left ventricular function, 2) prescription rate of angiotensin-converting enzyme inhibitor or angiotensin II receptor blockers (ACEI/ARB), 3) smoking cessation counseling, and 4) record of the hospital discharge instructions. However, the availability of care guidelines does not guarantee that HF management is standardized among the institutions. Also, little is known about the adherence of the institutions to these clinical practice guidelines and how the quality indicators, which reflect the degree of adherence to these guidelines, differ between the institutions.^3^ The implementation of a clinical practice protocol is an alternative to increase the adherence to quality indicators in HF. However, the real impact of the implementation of a protocol in clinical practice as analyzed by quality indicators has not yet been clearly established.

Thus, the aim of this study was to compare the adherence rates to the prescription of ACEI/ARB and beta-blocker at hospital discharge in a hospital that adopts case management to supervise the implementation of a clinical practice protocol and another hospital that follows treatment guidelines, both located in the city of Sao Paulo, Brazil.

## Methods

This prospective observational study compared the rates of ACEI/ARB and beta-blocker prescription at hospital discharge in patients hospitalized due to HF in two Brazilian hospitals, one with a care management program that supervises a clinical practice protocol (HCP), and another that only follows care guidelines (HCG), both located in the city of Sao Paulo, Brazil.

### Hospitals

The HCG is a public university hospital of high complexity specialized in cardiology, pulmonology, and cardiac and thoracic surgeries. Approximately 80% of the costs with care at the HCG are financed by the Brazilian unified health system *(Sistema Unico de Saude,* SUS). This hospital is a large teaching and research center, and a hub for health care ranging from disease prevention to treatment. The hospital has 629 beds distributed in seven inpatient units and six intensive care units (ICU). It also has research laboratories and a unit dedicated to complex diagnostic tests. This hospital has an annual average of 260 thousand medical consultations, 13 thousand admissions, 5 thousand surgeries, 2 million laboratory tests, and 330 thousand complex diagnostic tests.^5^

The HCP is a private and not-for-profit general hospital focused on the treatment of complex diseases. The hospital incorporates all the dimensions of health care, including disease promotion, prevention, diagnosis, treatment, and rehabilitation. This hospital offers care in several medical specialties, including cardiology. It has 647 beds distributed in inpatient, semi-intensive and coronary units, and ICU. In 2013, the hospital had approximately 36,857 surgical procedures, 50,311 hospitalizations and 5,413,834 diagnostic tests.

In 2006, the HCP implemented a clinical practice protocol for HF with nurse-supervised case management. The protocol was based on clinical care guidelines and information based on evidence, and aimed at standardizing the care of patients with HF.

The main aim of the case management is to analyze the quality indicators by following the patients included in the protocol from hospital admission to discharge. Information relevant to HF is then collected from the medical charts and organized in a database for analysis and preparation of reports.

### Population

We analyzed 1,052 patients consecutively admitted with a main diagnosis of HF functional class III/IV according to the New York Heart Association (NYHA) and left ventricular systolic dysfunction (left ventricular ejection fraction [LVEF] lower than or equal to 45%), between August 1st, 2006, and December 31st, 2008. Of these patients, 671 (64%) were seen at the HCP and 381 (36%) at the HCG.

At the HCP the information was collected in real time. Actions were then implemented based on the adherence to the clinical practice protocol. The following inclusion criteria that were defined for the clinical practice protocol at the HCP and were also applied for data collection at the HCG were valid for the present study:

- Age ≥18 years;
- Presence of documented systolic ventricular dysfunction (LVEF ≤ 45% or description in the medical chart of moderate to severe systolic dysfunction).

In addition to the above criteria, the patient should also have one of the following manifestations as a reason for admission:

- Acute HF (HF clinical syndrome without a prior diagnosis);
- Decompensated chronic HF (hospitalization for acute or gradual exacerbation of signs and symptoms in patients with a previous diagnosis of HF) or refractory (chronic low output, with or without signs of congestion).
- Cardiogenic shock;
- Acute pulmonary edema.

To analyze the indicator *rate of prescription of ACEI/ARB at hospital discharge,* we considered as eligible, according to the JCI criteria,^6^ those patients:

- Discharged from the hospital with less than 120 days from their admission;
- With an LVEF < 40%;
- Admitted directly to the hospitals (no transferences);
- Without description in the medical charts of palliative care;
- Who requested to be discharged from the hospital;
- Without registration in the medical charts of drug intolerance.

To analyze the indicator *prescription of beta-blocker at hospital discharge*, we considered as eligible those patients without contraindication to use the medication according to the guidelines.^7^

### Quality indicators

The indicators selected for the comparison between the hospitals were the rate of prescription of beta-blockers and the rate of prescription of ACEI/ARB, both analyzed at hospital discharge. The information on the prescription rates of these medications was collected from chart notes and prescriptions recorded within 24 hours from the discharge of the patient.

The formula used for the calculation of the rate of prescription of the indicators was the ratio between the number of patients with HF who were eligible to receive the medication and effectively received it over the total number of patients eligible to receive the medication multiplied by 100.

### Clinical outcome

In-hospital mortality data were collected from both hospitals and considered in the analysis.

### Statistical analysis

Numerical variables were presented as mean and standard deviation or median and interquartile variation. We used the Student's t-test or Mann-Whitney test for comparisons when appropriate.

Categorical variables were presented as absolute and relative frequencies and analyzed with the chi-square test.

To test the association of variables with the rate of prescription of beta-blocker and ACEI/ARB at hospital discharge, we used a logistic regression model adjusted for hospital type, gender, age, presence of permanent pacemaker and implantable cardioverter-defibrillator, left ventricular function, creatinine, heart rate, blood pressure, and history of chronic obstructive pulmonary disease, stroke, diabetes, and hypothyroidism.

To test the association of the variables with the in-hospital mortality rate we used a logistic regression model adjusted for the type of hospital, gender, age, presence of a permanent pacemaker, etiology of HF, blood pressure, heart rate, left ventricular function, creatinine, presence of anemia, history of chronic obstructive pulmonary disease, stroke, diabetes, hypothyroidism, and chronic renal failure. All tests were two-tailed, and the criterion for statistical significance was set at p<0.05. All analyzes were performed with the statistical program SPSS, version 20.0.

## Results

**Figure 1:**
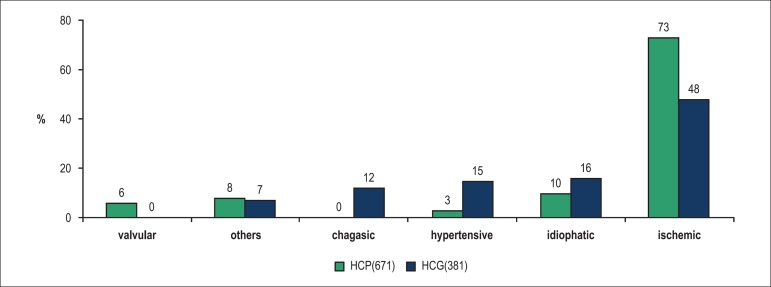
Comparison between heart failure etiologies in patients at the HCP and HCG. p < 0.001 for the comparison of the etiology frequency at both hospitals.

Patients at the HCG when compared with those at the HCP were younger and had more comorbidities. Table 1 shows the clinical characteristics of the patients according to the hospital. Figure 1 compares the prevalence of different etiologies between the hospitals. The predominant etiologies were ischemic (73%) at the HCP and ischemic, chagasic, and hypertensive at the HCG.

**Table 1:** Baseline clinical characteristics of the patients at both hospitals

**Characteristic**	**HCP (n = 671)**	**HCG (n = 381)**	**p**
Age, years	74.6 ± 12.1	63.7 ± 14.3	0.001
Male gender, n (%)	476 (71)	262 (69)	0.459
SBP, mmHg	126.2 ± 25.0	110.9 ± 10.0	0.001
DBP, mmHg	74.8 ± 16.3	70.2 ± 17.8	0.001
HR, bpm	84.1 ± 20.6	87.0 ± 13.0	0.01
LVEF, %	32.0 ± 7.7	28.0 ± 8.6	0.001
Creatinine, mg/dL	1.5 ± 1.1	1.9 ± 1.3	0.001
COPD, n (%)	50 (7)	39 (10)	0.119
Prior stroke, n (%)	84 (12)	45 (12)	0.737
Diabetes, n ( %)	237 (35)	169 (44)	0.004
CRF, n (%)	851 (12)	121 (32)	< 0.001
Permanent pacemaker, n (%)	159 (24)	56 (15)	< 0.001
Hypothyroidism, n (%)	115 (17)	121 (32)	< 0.001

HCG: hospital that follows treatment guidelines; HCP: hospital with a clinical practice protocol and case management; SBP: systolic blood pressure; DBP: diastolic blood pressure; HR: heart rate (in beats per minute); LVEF: left ventricular ejection fraction; COPD: chronic obstructive pulmonary disease; CRF: chronic renal failure.

### Quality indicators

The comparison between the institutions showed no difference in rates of beta-blocker prescription at discharge: HCP = 373/537 (69%) and HCG = 170/246 (69%), p = 0.458. There was also no difference in rates of ACEI/ARB prescription at hospital discharge: HCP = 213/257 (83%) and HCG = 141/163 (86%), p = 0.162.

In the adjusted model of logistic regression, we observed that the greater the age of the patients, the lower were their chances of receiving a beta-blocker prescription at hospital discharge at both institutions (Table 2). In contrast, lower LVEF and higher cardiac rate were associated with an increased chance of receiving the prescription at discharge (Table 2).

**Table 2:** Univariate and multivariate logistic regression analyses testing the association of different variables with the prescription of a beta-blocker at discharge.

**Variable**	**Univariate Analysis**			**Multivariate Analysis**		
	**OR**	**(95%) CI**	**p**	**OR**	**(95%) CI**	**p**
Male gender	1.32	(0.95 - 1.83)	0.095	1.19	(0.84 - 1.67)	0.328
COPD	0.82	(0.44 -1.53)	0.531			
Stroke	0.91	(0.58 - 1.42)	0.689			
DM	1.06	(0.78 - 1.45)	0.729			
Hypothyroidism	0.94	(0.65 - 1.35)	0.756			
Pacemaker	0.94	(0.65 - 1.36)	0.741			
ICD	0.81	(0.42 - 1.58)	0.540			
HCP	1.02	(0.74 - 1.41)	0.920	1.37	(0.96 - 1.97)	0.087
Age, years	0.98	(0.96 - 0.99)	0.001	0.98	(0.97 - 0.99)	0.002
SBP, mmHg	1.00	(0.99 - 1.01)	0.974			
DBP, mmHg	1.01	(0.99 - 1.01)	0.340			
HR, bpm	1.010	(1.00 - 1.01)	0.024	1.01	(1.00 - 1.02)	0.041
LVEF, %	0.97	(0.95 - 0.99)	0.011	0.98	(0.96 - 1.00)	0.023
Creatinine, mg/dL	1.07	(0.92 - 1.23)	0.351			

COPD: chronic obstructive pulmonary disease; DM: diabetes mellitus; ICD: implantable cardioverter-defibrillator; HCP: hospital with a case management program; SBP: systolic blood pressure; DBP: diastolic blood pressure; HR: heart rate (in beats per minute); LVEF: left ventricular ejection fraction.

As for the prescription of ACEI/ARB at hospital discharge, the presence of a pacemaker and lower LVEF showed association with a greater chance of prescription of ACEI/ARB (Table 3). In contrast, the occurrence of hypothyroidism was associated with a lower chance of prescription of one of these medications (Table 3).

**Table 3:** Univariate and multivariate logistic regression analyses testing the association of different variables with the use of ACEI/ARB at hospital discharge

**Variable**	**Univariate Analysis**			**Multivariate Analysis**		
	**OR**	**(95%) CI**	**p**	**OR**	**(95%) CI**	**p**
Male gender	1.08	(0.61 - 1.91)	0.783			
COPD	3.08	(0.72 - 13.19)	0.111	3.36	(0.74 - 15.26)	0.117
Stroke	0.87	(0.414 - 1.82)	0.706			
DM	1.77	(0.92 - 3.39)	0.082	1.54	(0.77 - 3.08)	0.225
Hypothyroidism	2.30	(1.00 - 5.25)	0.042	2.66	(1.09 - 6.47)	0.031
Pacemaker	0.34	(0.19 - 0.61)	< 0.001	0.37	(0.20 - 0.70)	0.002
ICD	0.71	(0.25 - 1.96)	0.564*			
HCP	0.74	(0.42 - 1.30)	0.289	1.05	(0.54 - 2.02)	0.893
Age, years	0.99	(0.42 - 1.30)	0.323			
SBP, mmHg	1.01	(0.42 - 1.30)	0.159	1.01	(0.99 - 1.03)	0.23
DBP, mmHg	1.01	(0.42 - 1.30)	0.128	1.00	(0.97 - 1.02)	0.711
HR, bpm	1.03	(0.42 - 1.30)	0.002	1.02	(1.00 - 1.04)	0.082
LVEF, %	0.95	(0.42 - 1.30)	0.012	0.94	(0.90 - 0.99)	0.019
Creatinine, mg/dL	0.38	(0.42 - 1.30)	0.008	0.49	(0.22 - 1.06)	0.068

COPD: chronic obstructive pulmonary disease; DM: diabetes mellitus; ICD: implantable cardioverter-defibrillator; HCP: hospital with a case management program; SBP: systolic blood pressure; DBP: diastolic blood pressure; HR: heart rate; LVEF: left ventricular ejection fraction.

### In-hospital mortality

The rate of in-hospital mortality at the HCP was 16.5% (n = 106/381) compared with 27.8% (n = 111/671) at the HCG (p < 0.001). In the adjusted logistic regression model, the implementation of a clinical practice protocol was independently associated with a lower mortality rate (odds ratio = 2.94, 95% confidence interval = 1.92-4.55, p = 0.001; Table 4).

**Table 4:** Univariate and multivariate logistic regression analyses testing the association of different variables with in-hospital mortality

**Variable**	**Univariate Analysis**			**Multivariate Analysis**		
	**OR**	**(95%) CI**	**p**	**OR**	**(95%) CI**	**p**
HCG	4.23	(2.97 - 6.03)	< 0.001	2.94	(1.92 - 4.55)	< 0.001
Age, years	0.99	(0.98 - 1.00)	0.073	1.02	(1.01 - 1.03)	0.004
Male gender	0.94	(0.66 - 1.36)	0.759			
Ischemic CHF	0.80	(0.57 - 1.12)		0.96	(0.64 - 1.42)	
SBP, mmHg	0.97	(0.96 - 0.97)	< 0.001	0.98	(0.97 - 0.98)	< 0.001
DBP, mmHg	0.96	(0.95 - 0.97)	< 0.001	0.99	(0.97 - 1.00)	0.117
HR, bpm	1.01	(1.00 - 1.02)	0.020	1.02	(1.00 - 1.02)	0.002
LVEF, %	0.95	(0.93 - 0.97)	< 0.001	0.97	(0.95 - 0.99)	0.028
Creatinine, mg/dL	1.30	(1.16 - 1.46)	< 0.001	1.21	(1.06 - 1.38)	0.004
COPD	1.80	(1.07 - 3.03)	0.025	1.84	(1.02 - 3.34)	0.043
Stroke	0.75	(0.43 - 1.31)	0.314			
Diabetes	1.37	(0.98 - 1.92)	0.066	1.24	(0.84 - 1.82)	0.274
CRF	2.52	(1.74 - 3.64)	< 0.001			
Hypothyroidism	1.63	(1.12 - 2.36)	0.01	1.35	(0.88 - 2.06)	0.164
Anemia	1.34	(0.68 - 2.62)	0.392			
Permanent pacemaker	0.71	(0.45 - 1.11)	0.134	0.66	(0.40 - 1.10)	0.112

HCG: hospital that follows treatment guidelines; CHF: congestive heart failure; SBP: systolic blood pressure; DBP: diastolic blood pressure; HR: heart rate (in beats per minute); LVEF: left ventricular ejection fraction; COPD: chronic obstructive pulmonary disease; CRF: chronic renal failure.

## Discussion

The main findings of this study were that 1) the rates of beta-blocker and ACEI/ARB prescription at hospital discharge were similar in both institutions, and 2) in-hospital mortality was lower at the HCP

The implementation of the protocol through case management at the HCP imposed a professional challenge to the managing nurse due to the open medical staff of the institution. As described in previous publications, the skills required from the nurse improved over time, including assessment, planning, implementation, coordination, and monitoring of therapeutic options.^8,9^

The clinical practice protocol established at the HCP has not yet reached a mature stage, which may explain the similar rates of ACEI/ARB and beta-blocker prescription at discharge at both hospitals. Makdisse et al.^10^ have shown that the implementation of a clinical practice protocol undergoes different phases of development: pre-implementation (around 2 years), maturation (around 3 years), and protocol establishment (5 years or more after implementation). In these phases, the adherence to the quality indicators tends to improve with time through constant approaches and direct actions to reinforce the protocol.^10^ In fact, the achievement of an establishment phase in a protocol seems to be an important factor to increase the adherence to the medications. As demonstrated in the ADHERE study that analyzed more than 280 million data from *Medicare* and *Medicaid* beneficiaries, the use of oral medications to treat HF, such as beta-blockers, increased over time.^11^

The data collection period of this study was 3 years. This short period hinders a comparison of the results obtained in consecutive years within the same institution, but is long enough to compare the two institutions. Although hospital accreditation organizations do not consider the prescription of beta-blockers as a gold-standard quality indicator, there is evidence that the use of beta-blockers at hospital discharge with or without ACEI/ARB decreases the rates of mortality and hospital readmission between 60 and 90 days after discharge.^12,13^ In elderly patients, this initiative decreases the mortality and readmission rates for any cause during 4 years of follow-up.^14,15^ In addition, the study *Carvedilol ACE-Inhibitor Remodeling Mild CHF Evaluation* (CARMEN), carried out in 67 centers in 13 European countries, also pointed out that the use of beta-blockers associated with ACEI produced more favorable effects in reversing left ventricular remodeling. The CARMEN study also showed that these drugs add valuable contributions to the clinical condition and life expectancy of the patient.^13^

We selected the prescription of ACEI/ARB at hospital discharge as a quality indicator since these medications are selected by care guidelines and accreditation agencies such as the JCI for their robust scientific evidence on mortality reduction in patients with HF.^16-18^

Both institutions analyzed in this study, regardless of adopting a protocol, follow recommendations of the best available scientific evidence. Since the HCG is linked to a university, the medical decisions in this institution are based on guidelines and academic decisions. As for the HCP the professional in charge of case management takes a synergistic approach to standardize the practice based on the guidelines. Another issue that may be raised is the possibility that part of the clinical staff may work in both institutions, which would justify similar approaches in both hospitals. Although we have no information regarding the medical staff at the HCG, we speculate that this fact may have contributed in part to the similar findings. Although we found similar rates of medication adherence between the hospitals, we hope that this study can be used as a resource to evaluate the implementation of guidelines in clinical practice. As other previous studies, we hope this also offer insight for professionals assessing the quality of the treatment of cardiovascular diseases.^10,19-21^

We can infer that the HCP benefited from the protocol. The case management approach increased the chance to identify reasons why the medications were not prescribed. Also, the prescription rate of ACEI/ARB could have been lower in the absence of the protocol.^14,17,22^

The difference in rates of in-hospital mortality between the institutions cannot be attributed only to the implementation of the protocol at the HCP but also to different clinical characteristics of the patients in both institutions. Although the patients at the HCG were younger and had more advanced stages of the disease, they also presented higher rates of hypotension and cardiorenal syndrome. The logistic regression model used in this study was not sensitive enough to capture the distinctive feature between the populations. This hindered the minimization of the impact of the result in mortality rates between the institutions. This analysis, therefore, should have been more accurate to produce a more precise result.

In another analysis, we will discuss in greater depth the many benefits and adverse effects of drug therapy during hospitalization and its impact on long-term outcomes.

## Conclusion

There was no difference in prescription rates of beta-blocker and ACEI/ARB at hospital discharge between the institutions. There was lower in-hospital mortality at the HCP The difference in mortality may be attributed to distinct clinical characteristics of the patients in both hospitals.
